# Image Evaluation of the High Resolution VUV Spectrometer at SURF II by Ray Tracing

**DOI:** 10.6028/jres.103.029

**Published:** 1998-10-01

**Authors:** N. C. Das, R. P. Madden, H. M. Seyoum

**Affiliations:** Spectroscopy Division, Bhabha Atomic Research Center, Bombay2400 085, India; National Institute of Standards and Technology, Gaithersburg, MD 20899-0001; Department of Physics, University of the District of Columbia, Washington, DC 20008

**Keywords:** high resolution, off-plane Eagle, ray tracing, spectrometer, SURF II

## Abstract

A high resolution VUV spectroscopic facility has been in use for several years at SURF II, the Synchrotron Ultraviolet Radiation Facility at the National Institute of Standards and Technology in Gaithersburg, Maryland. At this facility, a combination of three cylindrical mirrors is utilized to focus the light originating in the storage ring onto the horizontal entrance slit of the spectrometer. The spectrometer uses a 6.65 m concave grating having a groove density of 4800 lines/mm in the off-plane Eagle mounting. In preparation for the installation of an array detector in the exit image plane, a ray tracing program has been formulated and spot diagrams have been constructed by plotting the coordinates of the points of intersection of the diffracted rays with the image plane, which is tangent to the Rowland circle. In creating the spot diagrams, we have considered both parallel and tilted configurations of the entrance slit with respect to the grating grooves. It is shown that the line widths of the spectral images can be reduced when the entrance slit is properly tilted. Finally, we have estimated the spectral widths of the images when they are recorded on an array detector placed tangent to the Rowland circle. We conclude that an image spectral width of 0.41 pm to 0.88 pm in first order can be achieved over the wavelength region of 40 nm to 120 nm.

## 1. Introduction

A series of publications [1–3] has recorded the construction details of the high resolution vacuum UV spectroscopic facility at SURF II, the Synchrotron Ultraviolet Radiation Facility, located at the National Institute of Standards and Technology in Gaithersburg, Maryland. This system consists of three cylindrical mirrors and a 6.65 m concave grating spectrometer in which the grating is used in the off-plane Eagle [4, 5] mounting. Light originating from the electron storage ring is focused by the cylindrical mirrors onto the horizontal entrance slit of the spectrometer. The spectrum is scanned by moving the horizontal exit slit vertically, in the direction of dispersion along the Rowland circle. In a previous paper [6], we discussed the imaging and the scanning principles of the spectrometer and the advantages of replacing the movable exit slit by an electronic array detector, the center of which would remain tangent to the Rowland circle for any scanning position of the grating. Using analytic equations involving approximations that neglect terms above fourth order, we computed the scanning parameters, the spectral resolution due to point and line conditions of entrance slit illumination, and the optimized tilts and curvatures of both the entrance slit and the exit slit. It was established that the resolution of the spectrometer with point source illumination of the entrance slit is somewhat superior to that with line source illumination.

In the practical situation, however, the grating is illuminated by an extended source whose effective size is limited by the length and breadth of the entrance slit, and therefore a ray trace of the spectrometer was desired. Initially, the popular ray trace program, Shadow [7], was investigated, but it proved unable to handle the off-plane geometry of the Eagle mounting. Thus, following Spencer and Murty [8], a ray tracing scheme has been formulated for the system. Using the ray tracing equations, a large number of rays which originate from the entire area of the entrance slit and illuminate the full aperture of the concave grating have been traced, and the coordinates of the points of intersection of the resulting diffracted rays with the detector plane have been computed. The detector plane is considered to be always tangent to the Rowland circle at the wavelength of interest. Finally, these coordinates of the ray intersection points have been plotted to construct the spot diagram which represents the spectral image. From the spot diagram, the blur of the spectral images has been estimated, and the line width as well as the resolution at various wavelengths has been computed. This ray tracing scheme treats the entrance slit as uniformly illuminated, which is a good approximation of the actual illumination over the area of the entrance slit for our system due to the substantial aberrations in the collection optics. Since rays to or from points representing the entire grating surface are included, an upper limit of abberation in the image should be obtained.

## 2. Ray Tracing Scheme

**Fig. 1:**
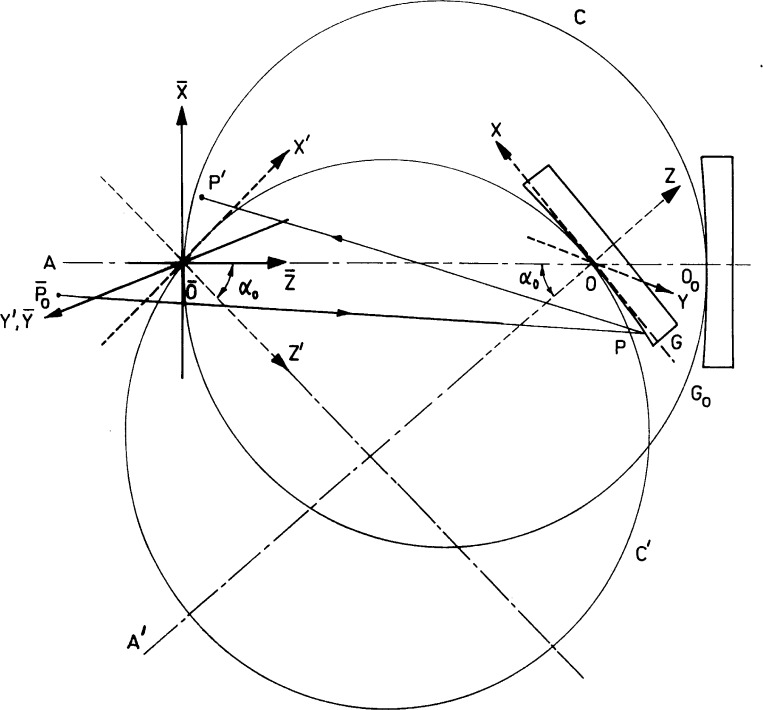
Diagram showing the grating configurations, ray paths and coordinate systems for the off-plane Eagle mounting of the concave grating.

Figure 1 shows the ray paths and the coordinate systems for two configurations of the concave grating when mounted in the off-plane Eagle mounting. G_0_ is the zero order position of the concave grating and $\bar{O}$ is the point of intersection of the grating axis with the Rowland circle.

Taking $\bar{O}$ as the origin, we have set up the ( $\bar{X}$, $\bar{Y}$, $\bar{Z}$,) system of coordinates in which the $\bar{Z}$ axis is coincident with the grating axis. The $\bar{X}$ axis is tangent to the Rowland circle C at the point $\bar{O}$ and the $\bar{Y}$ axis is perpendicular to the Rowland plane which is the plane of the paper. Thus the $\bar{Y}$ axis is also parallel to the direction of the grating grooves.

**Fig. 2:**
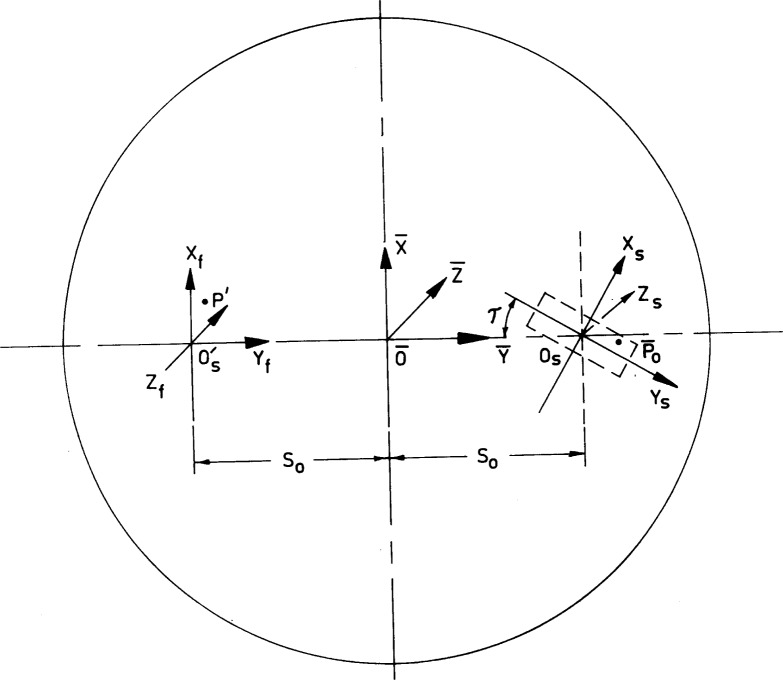
Diagram showing the end view of the spectrometer with three coordinate systems when observed from the entrance slit side. Note that the entrance slit is tilted by an angle *τ* with respect to the ruling direction ( $\bar{O}$, $\bar{Y}$).

The entrance slit is located on the ( $\bar{X}$, $\bar{Y}$) plane which is tangent to the Rowland circle C at the point $\bar{O}$. The orientation of the entrance slit with respect to the Rowland plane ( $\bar{X}$, $\bar{Z}$) and the $\bar{Y}$ axis is shown in Fig. 2, which represents an end-on view of the spectrometer slit plane when looking toward the grating. O_s_ is the central point of the entrance slit and is displaced from the Rowland plane ( $\bar{X}$, $\bar{Z}$) by a finite amount along the $\bar{Y}$ axis. The position of any point ${\bar{P}}_{0}$ on the entrance slit is described by the (*X*_s_, *Y*_s_, *Z*_s_) system of coordinates. In this coordinate system, the *X*_s_ axis and the *Y*_s_ axis are parallel to the breadth and the length of the entrance slit respectively. Both the *X*_s_ axis and the *Y*_s_ axis are located on the ( $\bar{X}$, $\bar{Y}$,) plane and they are rotated from the $\bar{X}$ axis and the $\bar{Y}$ axis by an angle which is equal to the tilt of the entrance slit from the direction of the grating grooves ( $\bar{O}$, $\bar{Y}$). The *Z*_s_ axis is, however, parallel to the $\bar{Z}$ axis.

Referring to Figs. 1 and 2, when the grating is located at the zero order position G_0_, close meridional rays, which originate from the central point O_s_ of the entrance slit and get diffracted by the concave grating, meet the ( $\bar{X}$, $\bar{Y}$) plane at the point O_s_′. The point O_s_′ is the central point of the zero order image and is situated on the $\bar{Y}$ axis. The displacement of the central points O_s_ and O_s_′ from the Rowland plane ( $\bar{X}$, $\bar{Z}$) when measured along the $\bar{Y}$ axis are equal and opposite. In order to focus the central wavelength at the zero order image position O_s_′, the grating is translated along the axis AO_0_ (see Fig. 1) and is simultaneously rotated about an axis which passes through the pole O and is perpendicular to the Rowland plane (i.e., the plane of the paper). Thus G is the new position of the concave grating with Rowland circle C′ and axis A’O. It may be seen that the Rowland circle has rotated about the $\bar{Y}$ axis by an angle which is equal to the angle of rotation of the grating.

Referring to Fig. 1, we set up a coordinate system (*X*′, *Y*′, *Z*′) with origin at the point $\bar{O}$. The *X*′ axis is tangent to the Rowland circle C′ at the point $\bar{O}$ and the *Y*′ axis is coincident with the $\bar{Y}$ axis. Thus, corresponding to the new position of the concave grating G, the (*X*′, *Y*′) plane is the image plane which is tangent to the Rowland circle C′ at the point $\bar{O}$. The *Z*′ axis is perpendicular to the (*X*′,*Y*′) plane at the point $\bar{O}$. Both the *X*′ axis and the *Z*′ axis are rotated from the $\bar{X}$ axis and the axis $\bar{Z}$ respectively by an angle which is equal to the rotation angle of the grating. In order to specify the position of any point *P* on the grating surface G, we have set up the (*X*, *Y*, *Z*) system of coordinates with origin at the pole 0 of the grating. In this coordinate system both the *X* axis and the *Y* axis are tangent to the grating surface at the pole 0 and the *Z* axis coincides with the axial direction A’0 of the grating. Also, the *Y* axis is parallel to the ruling direction which is perpendicular to the plane of the paper.

Referring to Figs. 1 and 2, we consider a typical incident ray ${\bar{P}}_{0}P$ originating from any point ${\bar{P}}_{0}$ on the entrance slit and meeting the grating surface G at the point *P*. The incident ray ${\bar{P}}_{0}P$ is diffracted in the direction *PP*′ and meets the image plane (*X*′, *Y*′) at the point *P*′. In order to specify the coordinates of the ray intersection point *P*′ on the image plane, we have set up the (*X*_f_, *Y*_f_, *Z*_f_) system of coordinates (see Fig. 2) having origin at the point $O_{s}^{\prime }$. The *Y*_f_ axis is coincident with the direction of the *Y*′ axis and the $\bar{Y}$ axis, the *X*_f_ axis is parallel to the *X*′ axis and the *Z*_f_ axis is parallel to the *Z*′ axis. Thus the image plane (*X*_f_, *Y*_f_) is coincident with the (*X*′, *Y*′) plane which is tangent to the Rowland circle C′ at the point $\bar{O}$.

Referring to Figs. 1 and 2, we now specify the geometrical parameters of the spectrometer as described below:

*R*: radius of curvature of the concave grating (diameter of the Rowland circle).
*d*_0_: grating groove spacing when measured on a plane which is tangent to the grating surface at the pole.
*L*_g_: length of the grating grooves (measured horizontally).
*W*_g_: width of the grating (measured vertically).
*α*_0_: angle of rotation of the grating normal A’0 from its original direction A0_0_.
*L*_s_: length of the entrance slit measured along the direction of the *Y*_s_ axis.
*W*_s_: width of the entrance slit measured along the direction of the *X*_s_ axis.
*τ*: tilt angle of the entrance slit from the groove direction ( $\bar{O}$, $\bar{Y}$). The sign of *τ* is positive when the entrance slit is rotated in the clockwise sense from the grating groove direction.
*S*_0_: distance of the central points of the entrance slit and the zero order image from the Rowland plane ( $\bar{X}$, $\bar{Z}$).
λ_0_: central wavelength which is defined as that which is focused at the zero order image position Os′ for any scanning position of the grating.
λ: any wavelength which is focused above or below the central wavelength image at Os′.
(*X*_s_, *Y*_s_, *Z*_s_): coordinates of any point ${\bar{P}}_{0}$ on the entrance slit.
(*X*,*Y*,*Z*): coordinates of any point *P* on the grating surface.

The central wavelength λ_0_ and the grating rotation angle *α*_0_ are related by [5]

$$2\sin\alpha_{0}{\left[1+S_{0}^{2}/R^{2}\cos^{2}\alpha_{0}\right]}^{–1/2}=n\lambda_{0}/d_{0},$$ (1)

where *S*_0_, *R*, and *d*_0_ are defined above and *n* is the diffraction order. This equation can be solved for sin *α*_0_ yielding

$$\begin{array}{l} \sin\alpha_{0}=(1/\sqrt{2})\{1+{\left[n\lambda_{0}/2d_{0}\right]}^{2}-\{{\left(1-{\left[n\lambda_{0}/2d_{0}\right]}^{2}\right)}^{2}\\ -{\left(n\lambda_{0}S_{0}/d_{0}R\right)}^{2}{\}}^{1/2}{\}}^{1/2}. \end{array}$$ (2)

Following Spencer and Murty [8], the ray tracing equations of the spectrometer have been derived as follows:

- Coordinates of the point ${\bar{P}}_{0}$ with respect to the ( $\bar{X}$, $\bar{Y}$, $\bar{Z}$) system:
  $$\begin{array}{l} {\bar{X}}_{0}=X_{s}\cos\tau –Y_{s}\sin\tau \\ {\bar{Y}}_{0}=X_{s}\sin\tau +Y_{s}\cos\tau +S_{0}\\ {\bar{Z}}_{0}=Z_{s} \end{array}$$ (3)
- Coordinates of the origin 0 with respect to the ( $\bar{X}$, $\bar{Y}$, $\bar{Z}$) system:
  $${\bar{x}}_{0}=0;{\bar{y}}_{0}=0;{\bar{z}}_{0}=–R\cos\alpha_{0}$$ (4)
- Coordinates of the point ${\bar{P}}_{0}$ with respect to the (*X*, *Y*, *Z*) system:
  $$\begin{array}{l} X_{0}=\left({\bar{X}}_{0}-{\bar{x}}_{0}\right)\cos\alpha_{0}-\left(\bar{Z}-{\bar{z}}_{0}\right)\sin\alpha_{0}\\ Y_{0}={\bar{Y}}_{0}-{\bar{y}}_{0}\\ Z_{0}=\left({\bar{X}}_{0}-{\bar{x}}_{0}\right)\sin\alpha_{0}+\left({\bar{Z}}_{0}-{\bar{z}}_{0}\right)\cos\alpha_{0} \end{array}$$ (5)
- Relation between the coordinates of the point *P* on the grating surface (noting that *R* is negative):
  $$Z=R+{\left[R^{2}-\left(X^{2}+Y^{2}\right)\right]}^{1/2}$$ (6)
- Path length of the incident ray ${\bar{P}}_{0}P\equiv S:$
  $$S={\left[{\left(X-X_{0}\right)}^{2}+{\left(Y-Y_{0}\right)}^{2}+{\left(Z-Z_{0}\right)}^{2}\right]}^{1/2}$$ (7)
- Direction cosines of the incident ray ${\bar{P}}_{0}P$ in the *X*, *Y*, *Z* system of coordinates:
  $$\begin{array}{l} k=\left(X-X_{0}\right)/S\\ l=\left(Y-Y_{0}\right)/S\\ m=\left(Z-Z_{0}\right)/S \end{array}$$ (8)
- direction cosines of the normal at the point *P* in the X, Y, Z system of coordinates:
  K=–X/R;L=−Y/R;M=1−(Z/R) (9)
- Direction cosines of the diffracted ray *PP'* in the *X*, *Y*, *Z* system of coordinates:
  $$\begin{array}{l} k^{\prime }=k-\Lambda u+T_{f}K\\ l^{\prime }=l-\Lambda v+T_{f}L\\ m^{\prime }=m-\Lambda w+T_{f}M \end{array}$$ (10)
  Λ=nλ/d (11)
  In Eq. (11)*d* is the local grating spacing at any point *P* on the ruled surface of the concave grating, *n* is the diffraction order, and *λ* is the diffracted wavelength. The local grating spacing, *d*, is measured along the curved surface of the concave grating and it is slightly different from the conventional grating spacing, *d*_0_, which is measured along a plane tangent to the grating surface at the pole. Thus for a conventional concave grating having uniform grating spacing, *d*_0_, the local grating spacing, *d*, is identical to *d*_0_ only at the pole and it varies throughout the entire ruled surface of the grating.
  Equations (10) are simplified forms of the three dimensional diffraction equations of Spencer and Murty [8] when one considers a reflection grating. In these equations (*K*,*L*,*M*) are the direction cosines of the normal to the grating surface at the point of incidence. The significance of the parameters (*u*,*v*,*w*) and the multiplier, *T*_f_, in Eqs. (10) are as follows: While deriving the three dimensional diffraction equations of any grating from the vector form of the diffraction equation, Spencer and Murty [8] have considered three orthogonal unit vectors at the incident point of the grating surface. Of these three unit vectors, the first is in the direction of the normal to the grating surface, the second is parallel to the grooves, and the third is perpendicular to the grooves at the point of incidence. The components of the above mentioned first and third unit vectors in the (*X*,*Y*,*Z*) directions have been specified by (*K*, *L*, *M*) and (*u*, *v*, *w*) respectively. These six components of the unit vectors describe the orientation of the grooves on the grating surface. Following Spencer and Murty [8], for a conventional concave reflection grating where the grooves are located at the intersection of equispaced parallel planes with the spherical surface, the groove parameters (*u*,*v*,*w*) and the local grating spacing are given by:
  $$\begin{array}{l} u=1/{\left[1+K^{2}/\left(L^{2}+M^{2}\right)\right]}^{1/2}\\ v=-KLu/\left(L^{2}+M^{2}\right)\\ w=-KMu/\left(L^{2}+M^{2}\right) \end{array}$$ (12)
  $$d=d_{0}/\left|u\right|.$$ (13)
  In our case, physically meaningful results require the use of the negative square root in the equation for *u* above.
  As discussed by Spencer and Murty [8], one can obtain a quadratic equation in *T*_f_ by squaring and adding Eqs. (10), and equating to unity. In this quadratic equation, if the coefficient of the squared term is set to unity, then the coefficient of the linear term is 2*a* and the coefficient of the constant term is *b*′ where *a* and *b*′ are given by:
  $$a=\left(kK+lL+mM\right)/\left(K^{2}+L^{2}+M^{2}\right)$$ (14)
  $$b\prime =\left[\Lambda^{2}-2\Lambda \left(ku+lv+mw\right)\right]/\left(K^{2}+L^{2}+M^{2}\right).$$ (15)
  In order to solve the quadratic equation in *T*_f_, Spencer and Murty [8] have used the Newton-Raphson iteration technique and have expressed the iteration formula as:
  $$T_{n+1}=\left(T_{n}^{2}-b\prime \right)/2\left(T_{n}+a\right)$$ (16)
  where *T*_f_ = limit of *T_n_* as *n* → ∞.
  When using the iteration Eq. (16) for a reflection grating, the first approximation of *T_n_* can be assumed to be
  $$T_{1}=\left(b\prime /2a\right)-2a.$$ (17)
  With this first approximation, a highly accurate value for *T*_f_ is obtained after only four or five iterations.
- Coordinates of the point *P* in the ( $\bar{X}$, $\bar{Y}$, $\bar{Z}$) system:
  $$\begin{array}{l} \bar{X}=X\cos\alpha_{0}+Z\sin\alpha_{0}+{\bar{x}}_{0}\\ \bar{Y}=Y+{\bar{y}}_{0}\\ \bar{Z}=-X\sin\alpha_{0}+Z\cos\alpha_{0}+{\bar{z}}_{0} \end{array}$$ (18)
- Direction cosines of the diffracted ray *PP*′ in the ( $\bar{X}$, $\bar{Y}$, $\bar{Z}$) system of coordinates:
  $$\begin{array}{l} \bar{k}\prime =k\prime \cos\alpha_{0}+m\prime \sin\alpha_{0}\\ \bar{l}\prime =l\prime \\ \bar{m}\prime =-k\prime \sin\alpha_{0}+m\prime \cos\alpha_{0} \end{array}$$ (19)
- Coordinates of the point *P* in the (*X*′, *Y*′, *Z*′) system:
  $$\begin{array}{l} X\prime =\bar{X}\cos\alpha_{0}+\bar{Z}\sin\alpha_{0}\\ Y\prime =\bar{Y}\\ Z\prime =-\bar{X}\sin\alpha_{0}+\bar{Z}\cos\alpha_{0} \end{array}$$ (20)
- Direction cosines of the diffracted ray *PP*′ in the (*X*′, *Y*′, *Z*′) system of coordinates:
  $$\begin{array}{l} k_{d}^{\prime }=\bar{k}\prime \cos\alpha_{0}+\bar{m}\prime \sin\alpha_{0}\\ l_{d}^{\prime }=\bar{l}\prime \\ m_{d}\prime =\;–\bar{k}\prime \sin\alpha_{0}+\bar{m}\prime \cos\alpha_{0} \end{array}$$ (21)
- Coordinates of the ray intersection point *P*′ in the (*X*′, *Y*′, *Z*′) system:
  $$\begin{array}{l} X_{p}\prime =X\prime +k_{d}\prime S\prime \\ Y_{p}\prime =Y\prime +l_{d}\prime S\prime \\ Z_{p}\prime =Z\prime +m_{d}\prime S\prime \end{array}$$ (22)
  $$\text{where}\;S\prime =-Z\prime /m_{d}\prime$$ (23)
- Coordinates of the ray intersection point *P*′ in the (*X*_f_, *Y*_f_, *Z*_f_) system:
  $$X_{f}=X_{p}\prime ;Y_{f}=Y_{p}\prime +S_{0};Z_{f}=Z_{p}\prime .$$ (24)

## 3. Image Evaluation

The imaging properties of the SURF II high resolution VUV spectrometer have been evaluated by computer and the spot diagrams corresponding to various wavelengths plotted. For this purpose we have divided the entrance slit area into a grid structure consisting of a finite number of points at regular intervals along the length and the breadth of the entrance slit. Using the equations of the previous section, a large number of rays originating from each point of the grid structure and covering the full aperture of the concave grating have been traced and the coordinates of the ray intersection points on the image plane, which is tangent to the Rowland circle, have been determined. The geometrical parameters of the spectrometer used for calculations in the ray tracing equations are as follows:

$$\begin{array}{l} R=6.65\;m,L_{g}=110\;\text{mm},W_{g}=125\;\text{mm}\\ 1/d_{o}=4800{\;\text{mm}}^{-1},L_{s}=10\;\text{mm},W_{s}=10\mu m\\ \;\text{or}\;1.0\mu m.\\ S_{0}=84\;\text{mm},n=1. \end{array}$$

**Fig. 3:**
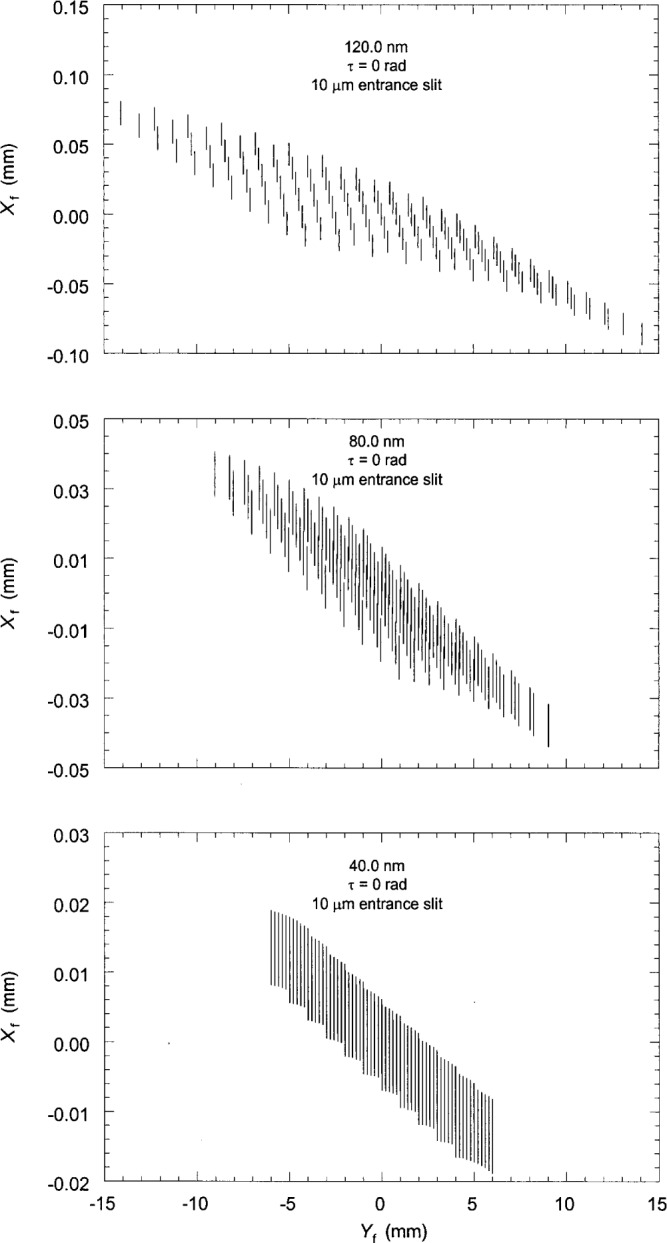
Spot diagrams in the image plane corresponding to central wavelengths of 40 nm, 80 nm, and 120 nm. The entrance slit length is 10 mm and the entrance slit width is 10 μm. The entrance slit tilt angle, *τ*, is zero. *X*_f_ and *Y*_f_ are the vertical and horizontal focal plane axes.

**Fig. 4:**
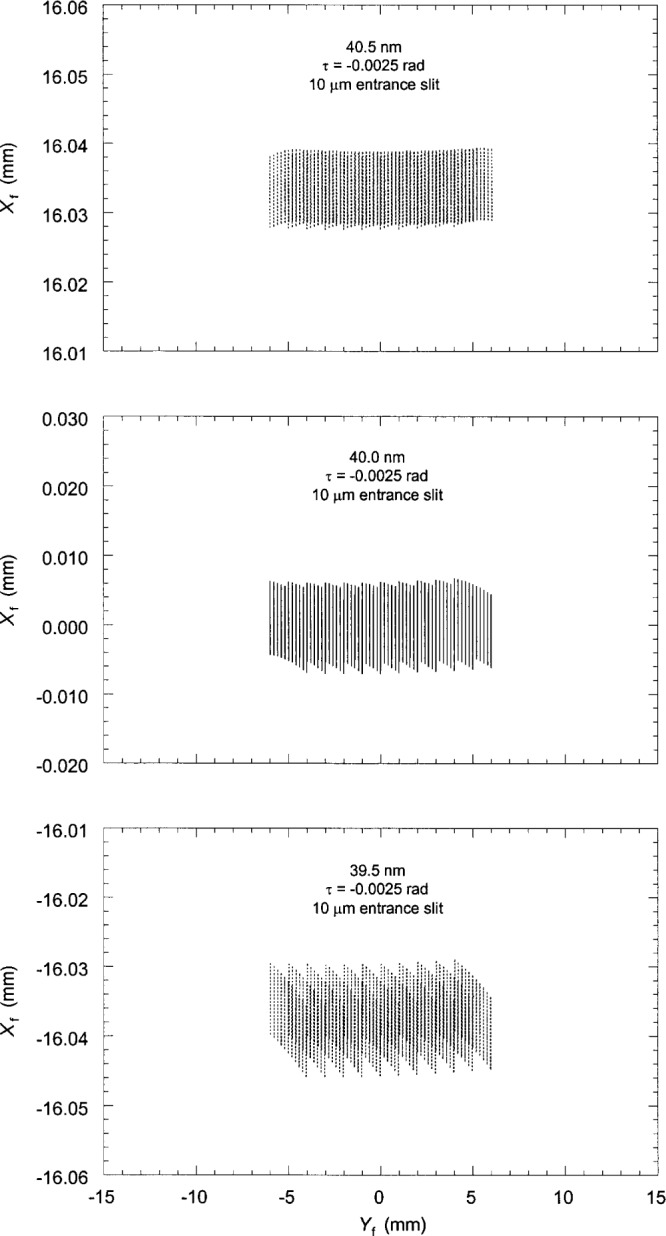
Spot diagrams in the image plane corresponding to a central wavelength of 40 nm and two outer wavelengths, which are 0.5 nm higher and 0.5 nm lower than the central wavelength. Note that the entrance slit tilt angle, *τ*, is – 0.0025 rad. The negative value of *τ* means that the entrance slit is rotated in the counter-clockwise sense from the ruling direction. The entrance slit length is 10 mm and the entrance slit width is 10 μm. *X*_f_ and *Y*_f_ are the vertical and horizontal focal plane axes.

**Fig. 5:**
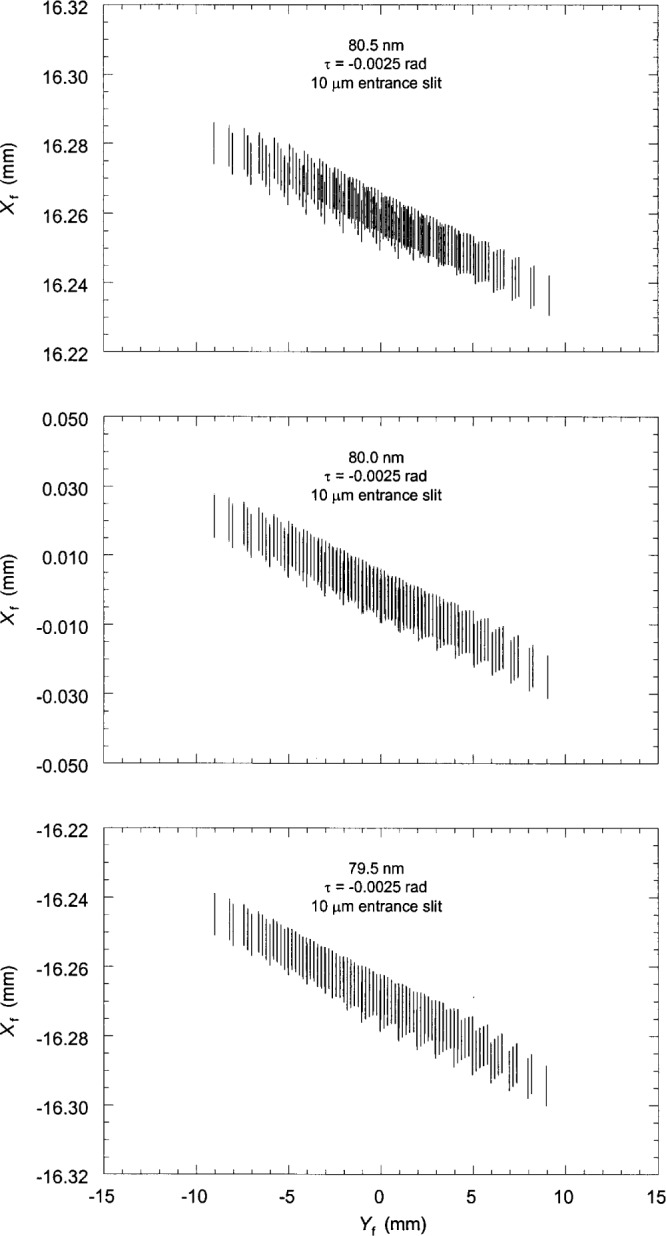
As for Fig. 4, except that the central wavelength is 80 nm.

**Fig. 6:**
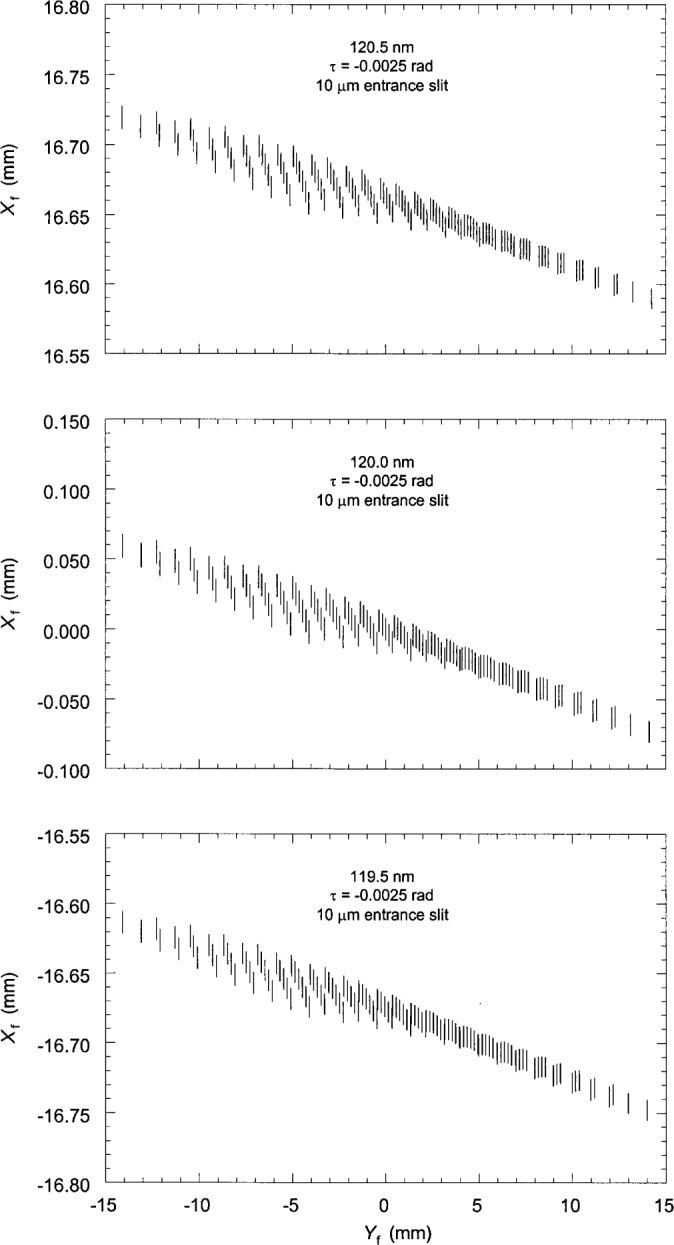
As for Fig. 4, except that the central wavelength is 120 nm.

Finally, the spot diagrams were constructed by plotting the coordinates of the ray intersection points. Figures 3 to 6 and Fig. 8 show spot diagrams for various parameter settings and wavelengths in the spectral region 40 nm to 120 nm. In each spot diagram the *Y*_f_ axis is parallel to the ruling direction of the concave grating, which is horizontal, and the *X*_f_ axis is the direction of dispersion, which is tangent to the Rowland circle and orthogonal to *Y*_f_. Thus the blur of the spectral images is measured by the spread of the spot diagram in the *X*_f_ direction.

Both the theoretical resolution, and the resolution calculated for this instrument in Ref. [6] for a 10 mm long entrance slit oriented at the optimum tilt angle, indicate optimum diffraction limited resolution at slit widths of 2 μm to 6 μm over the wavelength range 40 nm to 120 nm. In practice, we have found grating-limited resolution at somewhat wider slits, and therefore have calculated the resolution below for the more typical entrance slit width of 10 μm. Figure 3 shows the spot diagrams corresponding to the central wavelength of 40 nm, 80 nm, and 120 nm, calculated for an entrance slit width of 10 μm and an entrance slit length of 10 mm. The significance of the central wavelength has been discussed in Ref. [5] and the previous section. While plotting the spot diagrams of Fig. 3, it has been assumed that the entrance slit is parallel to the ruling direction so that the tilt angle *τ* is zero as labeled in the figures. It is to be noted from the above figures that, in general, the spectral lines are tilted and slightly curved even though the entrance slit is horizontal, and the line widths of the spectral images are not uniform throughout their entire length. However, as expected for the central wavelength, the mid-points of the spectral lines are zero on the horizontal axis, *Y*_f_.

Previous work [5, 6] has discussed the improvement (narrowing) of the spectral images when one uses an entrance slit which is tilted with respect to the ruling direction. Using analytic theory we have computed the optimized tilt of the entrance slit and it has been shown that the optimum tilt angle varies continuously with wavelength. Since it is not always possible in the practical situation to change the tilt angle of the entrance slit, we have chosen the optimum tilt angle corresponding to the central wavelength of 80 nm. Figures 4 to 6 show the spot diagrams corresponding to wavelengths of 40 nm, 80 nm and 120 nm for this tilt angle of the entrance slit, which is −0.0025 rad. A negative sign of the tilt angle, *τ*, as labeled on the spot diagrams indicates that the entrance slit is rotated in the counter-clockwise sense from the groove direction. As in Fig. 3, the spot diagrams of Figs. 4 to 6 have been calculated for an entrance slit width of 10 μm and an entrance slit length of 10 mm. In each of Figs. 4 to 6, the spot diagram in the center of the figure corresponds to the central wavelength, whereas the upper and lower spot diagrams correspond to two non-central wavelengths, which are 0.5 nm below and above the central wavelength.

It can be observed from the spot diagrams of Figs. 4 to 6 that the length of the spectral images increases with wavelength. When the spectrum around the central wavelength is scanned by moving the exit slit along the image plane in the direction of dispersion, one would like to use an exit slit sufficiently long to collect most of the flux in the spectral line. Increasing the length of the exit slit gives rise to a problem however. It may be seen that when the spectrum is scanned by moving a horizontal exit slit in the direction of dispersion, the effective line width of the spectral images is more than the actual line width since the spectral lines are, in general, tilted–even when using an optimized tilt of the entrance slit. In order to obtain the minimum line width while the spectrum is scanned by the exit slit, one would have to use a tilted exit slit, or as small a length as possible, the latter resulting in a loss of intensity. Tilting the exit slit would achieve improved resolution at the same intensity level; however, the angle of tilt is wavelength dependent. The situation is, however, different when the spectrum is recorded on an electronic array detector placed tangent to the Rowland circle and the readout of the array is processed by a computer. With this technique one can select the pixels to read out during data analysis which yield the minimum line width of the spectral images without suffering an intensity loss, and can record the minimum resolvable wavelength bandpass.

**Fig. 7:**
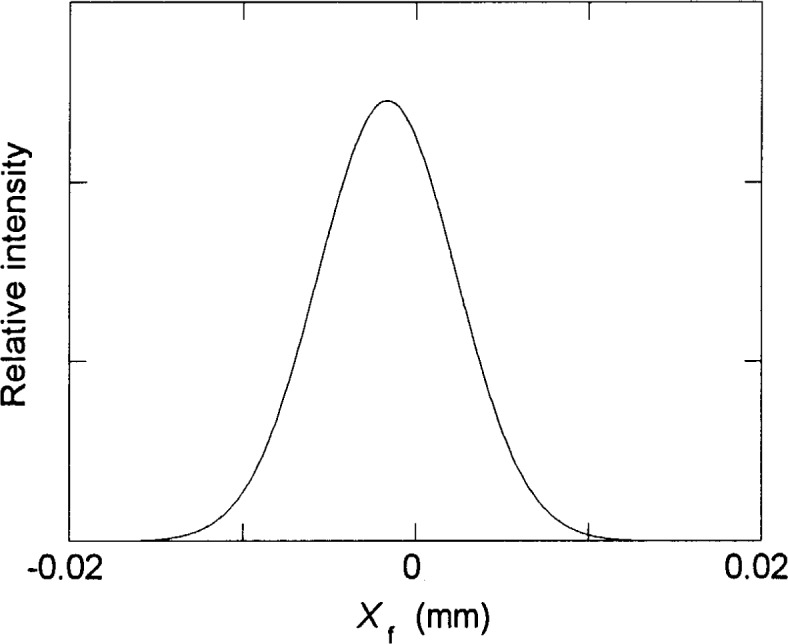
The slit function profile obtained by scanning a narrow (relative to the profile width) horizontal exit slit vertically through the spot diagram for a wavelength of 80 nm as shown in Fig. 5, with *τ* = – 0.0025 rad. The entrance slit length is 10 mm, the entrance slit width is 10 μm, and the length of the horizontal exit slit is 1 mm. *X*_f_ is the image plane vertical axis.

It is evident from Figs. 4 to 6 that the minimum spread of the spectral images in the vertical direction varies between 10 μm and 20 μm throughout the spectral region of interest. We can verify this by scanning a short exit slit in the dispersion direction (vertically in Figs. 4 to 6) through the spot diagrams as shown. Figure 7 shows the intensity distribution which one would obtain by scanning a very narrow (compared to the image width), 1 mm long horizontal exit slit through the image distribution shown in Fig. 5 for a wavelength of 80 nm. A gaussian slit function profile was used for the convolution, which is a reasonably approximate profile for slit widths set at 2 times the diffraction limit [9]. A half-maximum width of 9.4 μm was obtained from this analysis, approximating the ideal of 10 μm. Similar considerations lead to widths of 10 μm at 40 nm, broadening to 20 μm for a wavelength of 120 nm. The slight shift of the center of the profile in Fig. 7 from *X*_f_ = 0 is an artifact due to the restriction in the number of rays used for this calculation.

**Fig. 8:**
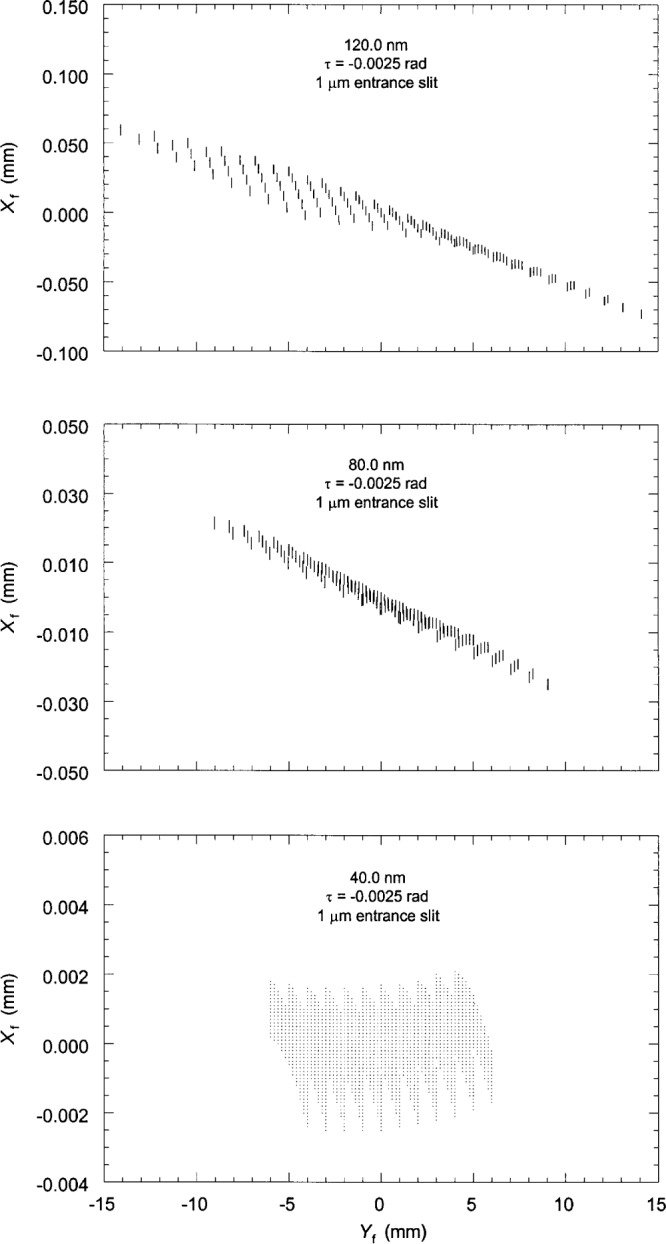
Spot diagrams in the image plane corresponding to central wavelengths of 40 nm, 80 nm and 120 nm for a narrower entrance slit than in Figs. 4 to 6. The entrance slit length is 10 mm, the entrance slit width is 1 μm, and the entrance slit tilt angle *τ* is −0.0025 rad.

**Fig. 9:**
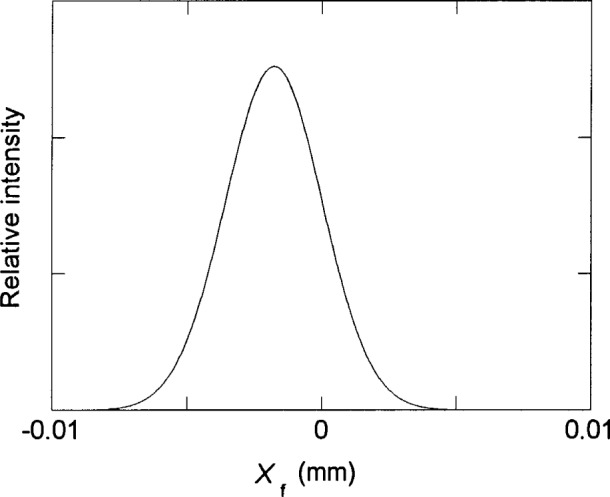
The slit function profile obtained by scanning a narrow (relative to the profile width) horizontal exit slit vertically through the spot diagram for a wavelength of 80 nm, as shown in Fig. 8. The entrance slit length is 10 mm, the entrance slit width is 1 μm, amd the length of the horizontal exit slit is 1 mm.

**Fig. 10:**
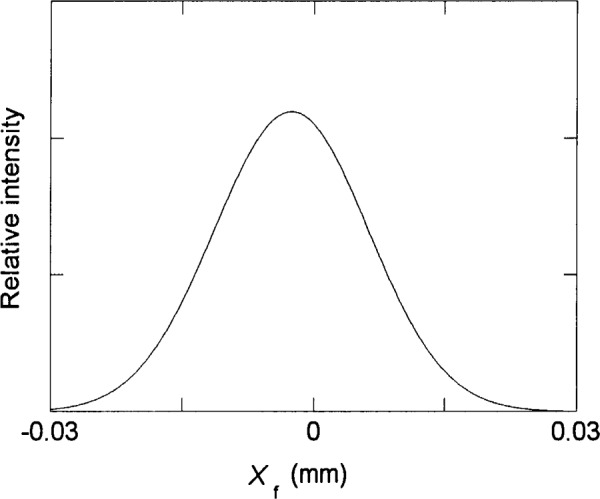
As in Fig. 9, except that the length of the horizontal exit slit used in the scan was 10 mm.

Figure 8 shows the spot diagrams corresponding to the central wavelengths of 40 nm, 80 nm and 120 nm for an entrance slit width narrowed, unrealistically, to 1 μm, maintaining an entrance slit length of 10 mm. These distributions are much narrower, and in fact are at the diffraction limit for 40 nm and 80 nm, while that for 120 nm shows only a modest improvement over that for a 10 μm entrance slit. For example, Fig. 9 shows the result of scanning a very narrow (compared to the image width), 1 mm long exit slit across the distribution shown in Fig. 8 for a wavelength of 80 nm. The indicated width is 4.3 μm. These image widths are, however, considerably larger than the 1 μm entrance slit, indicating the effects of aberration. Nevertheless, when a long exit slit is used the principal aberration is the tilt of the image. To quantify this effect, let us now scan the distribution using a longer horizontal exit slit. Figure 10 is the result obtained when a narrow exit slit (compared to the image width), 10 mm in length, is scanned in the dispersion direction (vertically) through the distribution shown in Fig. 8 for 80 nm. The profile width obtained is 20 μm, or nearly five times that for a 1 mm long exit slit (Fig. 9). Thus for a long exit slit, the narrower widths can only be achieved by using a tilted exit slit of variable tilt angle. However, the effect of a long slit can be obtained by appropriate interrogation of an array detector.

In conclusion, it appears that using a 10 μm wide, 10 mm long entrance slit tilted at the optimum angle for 80 nm, an ideal electronic array detector, and recording a narrow band of wavelengths around the central wavelength, the achievable exit image widths should be 10 μm at wavelengths 40 nm and 80 nm, and 20 μm at the wavelength 120 nm. Using the SURF II spectrometer dispersion of 0.0313 nm/mm, this implies spectral slit widths of 0.31 pm at 40 nm and 80 nm, and 0.63 pm at 120 nm, corresponding to first order spectral resolutions of 130 000, 260 000, and 190 000, respectively. We currently have on hand an array detector with pixels spaced at 7.5 μm and spacings of 5 μm are apparently now available. Thus the pixel density of presently available array detectors appears to be commensurate with this resolution.
